# Molecular Detection and Genotyping of *Coxiella*-Like Endosymbionts in Ticks that Infest Horses in South Korea

**DOI:** 10.1371/journal.pone.0165784

**Published:** 2016-10-28

**Authors:** Min-Goo Seo, Seung-Hun Lee, In-Ohk Ouh, Gwang Hyeop Lee, Youn-Kyoung Goo, Seungjoon Kim, Oh-Deog Kwon, Dongmi Kwak

**Affiliations:** 1 College of Veterinary Medicine, Kyungpook National University, Daegu, South Korea; 2 Animal and Plant Quarantine Agency, Gimcheon, South Korea; 3 Seowon Equine Clinic, Jeju, South Korea; 4 Department of Parasitology and Tropical Medicine, Kyungpook National University School of Medicine, Daegu, South Korea; 5 Cardiovascular Research Institute, Kyungpook National University, Daegu, South Korea; University of Kentucky College of Medicine, UNITED STATES

## Abstract

Members of the genus *Coxiella* can be transmitted from ticks to humans during contact with animals; *Coxiella* may thus spread from the infected horses or ticks to humans. In this study, the presence of *Coxiella burnetii* and *Coxiella*-like endosymbionts (CLE) in ticks found on infested horses was determined using PCR and genotyping. A total of 213 ticks were randomly collected from 51 horses (4–5 ticks per horse) raised on Jeju Island, Korea, between 2009 and 2013. All ticks were morphologically identified as adult *Haemaphysalis longicornis*, a predominant tick species widespread in Korea. Based on the results of nested PCR and 16S rRNA sequencing, CLE were detected in 121 (52.4%, 95% CI: 45.9–58.8) ticks. CLE 16S rRNA sequences from 9 randomly selected ticks were 100% identical. Phylogenetic analysis showed that these 9 sequences were highly similar (97.9–100%) to the sequences of clade B species, like the CLE previously described to be found in *Haemaphysalis* spp. This study showed that CLE are prevalent in ticks that infest horses reared on Jeju Island, and this is, to the best of our knowledge, the first study to describe CLE occurrence in ticks infesting animals reared in Korea. Because of the high prevalence of CLE in ticks found on horses, CLE transmission from ticks to other animals and humans remains a possibility. This warrants a detailed study of other hosts and regions. Considering the zoonotic potential of *Coxiella*, further strategic surveillance of *Coxiella* transmission is necessary.

## Introduction

Q fever, caused by *Coxiella burnetii*, is a zoonotic disease showing global distribution, and can cause serious complications in humans and animals [1]. When used as a biological weapon, *C*. *burnetii* may not cause high mortality, but it can induce an acute disabling disease [1]. *C*. *burnetii* is the only officially recognized species in the genus *Coxiella* [2], although another putative species (*C*. *cheraxi*) was reported in crayfish [3]. The phylogenetic origin of *C*. *burnetii* is unknown, but new *Coxiella*-like endosymbionts (CLE) have been isolated from ticks [4, 5]. CLE differ from *C*. *burnetii* in their biological features, and some may act as symbionts engaged in complicated interactions with ticks [5]. CLE are closely associated with, but genetically distinct from *C*. *burnetii*, suggesting that some diversity exists within the genus *Coxiella* [5].

The primary route of infection by *C*. *burnetii* in humans is ingestion or inhalation of the pathogen [6]. Although *Coxiella* has been isolated from several arthropods, primarily ticks, tick-borne transmission of Q fever in humans by tick bites or through feces is unlikely [7]. However, ticks can transmit *Coxiella* both transovarially and transstadially to their offspring, acting as a reservoir [6]. Infected ticks excrete high concentrations of *Coxiella* in feces, which contaminate the host skin. *Coxiella* has the ability to persist in the environment [6]. Therefore, ticks may be important for the environmental dissemination of *Coxiella*.

In field epidemiology, it is important to identify the specific tick species that carry this pathogen. More than 40 tick species are thought to be naturally infected with *C*. *burnetii* [6]. However, some strains, primarily visually identified as *C*. *burnetii*, might need to be reclassified as CLE [8]. Consequently, the statement that more than 40 tick species are infected by *C*. *burnetii* [6] may be incorrect, and should be revaluated when suitable molecular information is available [8]. Using multilocus DNA sequencing, the genus *Coxiella* has been classified into a minimum of 4 highly divergent genetic clades (A–D), with *C*. *burnetii* being categorized within clade A [5]. The genus *Haemaphysalis* consists of 166 different species and is the second largest tick genus in the family Ixodidae [9]. *Haemaphysalis longicornis* is a widespread tick species, predominant in Korea. Although relatively few published studies discuss the prevalence of tick-borne *Coxiella* in Korea, it is important to investigate tick infestation risk, especially from the zoonotic as well as public health perspective.

Recently, the horse industry in Korea has grown tremendously, thanks to the increase in global horse trade. About 25,819 horses have been raised in Korea in 2014; regionally, the highest percentage of horses (57.2%) was reared on Jeju Island, Korea [10]. Horseback riding can expose the rider to various ticks, and *Coxiella* may spread from the infected horses or ticks to humans [11]. Recently, in Korea, *C*. *burnetii* and CLE were detected in horses [12]. Therefore, the aim of this study was to use nested PCR (nPCR) and genotyping to investigate the occurrence of *C*. *burnetii* or CLE in vector ticks that infest horses on Jeju Island, Korea, and study their differences.

## Materials and Methods

### Ethics statement

The ticks were collected from horses during treatment or regular medical checkup, which did not require ethical approval from any authority; the same was true for undertaking this study. In addition, removal of ticks from horses is neither harmful nor against animal welfare. All procedures were performed by well-equipped veterinarians. This study did not involve endangered or protected species.

### Study area and tick collection

A total of 213 adult ticks were randomly harvested from 51 horses (4–5 ticks per horse) reared on Jeju Island between 2009 and 2013. The collected ticks were fixed in 70% ethanol until further use. Tick species and life stages were determined based on their morphological characteristics. For molecular analysis, ticks fixed in 70% ethanol were rinsed with distilled water and dried on sterile paper.

### DNA extraction and PCR analyses

The ticks were crushed with a sterile plastic homogenizer. Genomic DNA was extracted using the commercial DNeasy Blood and Tissue Kit (QIAGEN, Melbourne, Australia), according to the manufacturer’s instructions. The extracted DNA was stored at −20°C until further use. Commercial AccuPower HotStart PCR Premix Kit (Bioneer, Daejeon, Korea) was used for PCR amplification. nPCR was used to amplify the 16S rRNA of *C*. *burnetii* and CLE, and sequencing could differentiate between *C*. *burnetii* and CLE [5]. First-round PCR was performed with the primers Cox16SF1 (5′-CGTAGGAATCTACCTTRTAGWGG-3′) and Cox16SR2 (5′-GCCTACCCGCTTCTGGTACAATT-3′), which produced 1321–1429 bp amplicons. Then, nPCR was performed using the primers Cox16SF2 (5′-TGAGAACTAGCTGTTGGRRAGT-3′) and Cox16SR2, which produced 624–627 bp amplicons. Samples yielding amplicons of the expected size were bi-directionally sequenced using the primers Cox16SF1 and Cox16SR1 (5′-ACTYYCCAACAGCTAGTTCTCA-3′), which produced 719–826 bp amplicons. All PCR amplifications were performed using the Mastercycler Pro (Eppendorf, Hamburg, Germany), by using the following program: pre-denaturation at 93°C for 3 min; 30 cycles of denaturation at 93°C for 30 s, annealing at 56°C for 30 s, and polymerization at 72°C for 1 min; and a final post-polymerization step at 72°C for 5 min. PCR products of the second amplification process were analyzed by electrophoresis, with 10 μl of the reaction mixture and a 100 bp DNA ladder (Bioneer), by using 1.5% agarose gels, for 30 min at 100 V, and visualized using UV transillumination imaging after ethidium bromide staining.

### Phylogeny

Purified PCR products obtained using the specific primers (Cox16SF1 and Cox16SR1) were sequenced by Macrogen (Seoul, Korea). The nucleotide sequences were analyzed using the multiple sequence alignment program CLUSTAL Omega (ver. 1.2.1) [13] and corrected using BioEdit (ver. 7.2.5) [14]. Phylogenetic analysis was performed using MEGA (ver. 6.0) [15], following the maximum likelihood method. The aligned sequences were used for homology comparison of *Coxiella* 16S rRNA. The stability of the obtained trees was estimated by bootstrap analysis with 1,000 replicates.

### Statistical analysis

A 95% confidence interval (CI) for all estimates was calculated using Blaker’s method [16].

## Results

### Identification and PCR assay

All harvested ticks were identified as adult *H*. *longicornis*. Overall, 121 (52.4%, 95% CI: 45.9–58.8) of the 213 ticks tested positive for CLE by 16S rRNA nPCR (Table 1). Although tick-infested horses were not assessed in this study, 121 ticks carrying CLE were harvested from 42 (82.4%) of 51 horses.

**Table 1:** Prevalence of *Coxiella*-like endosymbionts in ticks infesting horses reared on Jeju Island, Korea, determined by 16S rRNA nested PCR.

Tick species	Developmental stage	Number of ticks tested	Number of ticks positive (%)	95% CI*
*Haemaphysalis longicornis*	Adult	231	121 (52.4)	45.9–58.8

*CI, confidence interval

### DNA sequencing and phylogenetic analysis

**Fig 1:**
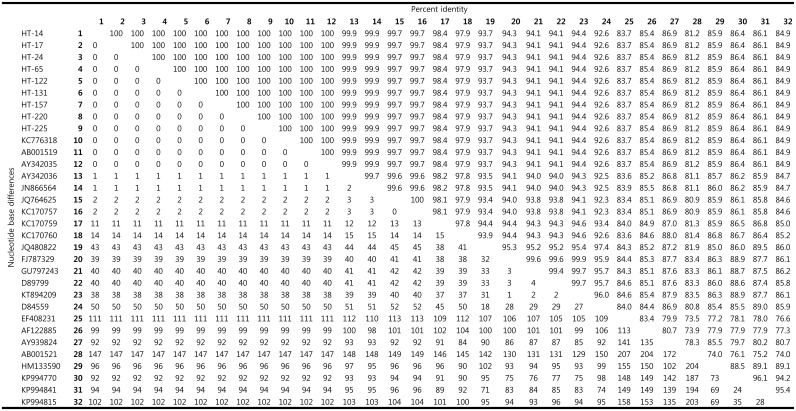
Comparison of *Coxiella* 16S rRNA nucleotide sequences. The upper matrix represents the percentage identity between *Coxiella* 16S rRNA sequences. The lower matrix represents the number of nucleotide base differences.

**Fig 2:**
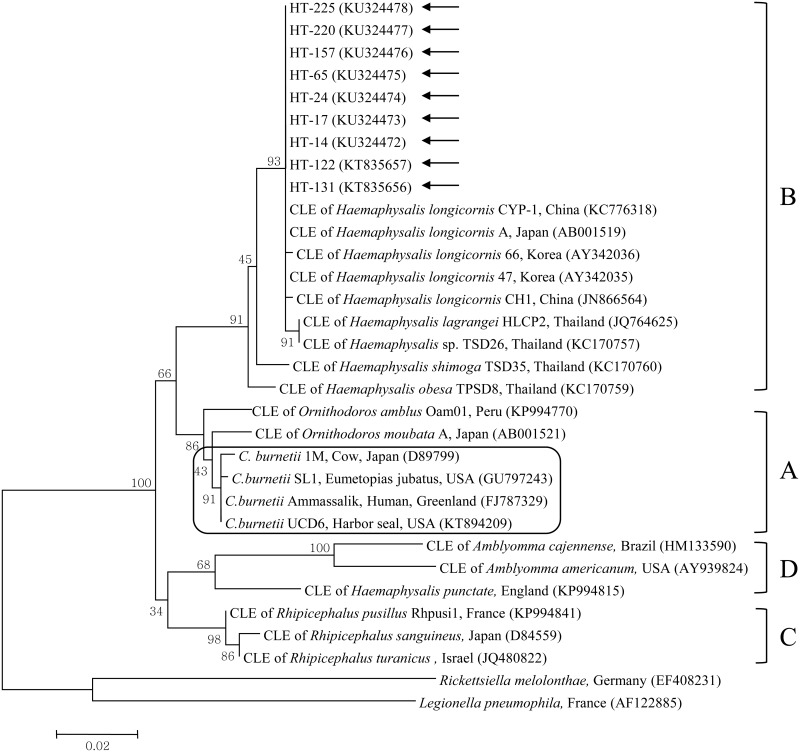
A phylogenetic tree constructed using the maximum likelihood method based on the 16S rRNA sequences of the genus *Coxiella*. *Coxiella*-like endosymbiont sequences analyzed in this study are marked by arrows. *C*. *burnetii* group is outlined within clade A. Four clades of the genus *Coxiella* are labeled A to D. The accession numbers of other sequences from GenBank are shown with the sequence names and countries. The numbers on the branches indicate bootstrap support (1,000 replicates). Scale bar shows the phylogenetic distance between sequences equivalent to 0.02 nucleotide substitutions per site. CLE = *Coxiella*-like endosymbionts.

Among the 121 nPCR-positive tick samples, 9 were randomly selected for subsequent nucleotide sequencing and phylogenetic analyses. The sequences obtained from these 9 ticks (HT-14, 17, 24, 65, 122, 131, 157, 220, and 225) were 100% identical (Fig 1) and have been deposited in GenBank with the accession nos. KU324472–KU324478 and KT835656–KT835657, respectively. The nucleotide sequences of these 9 samples showed high similarity (97.9–100%) to the species of clade B, similar to the CLE previously described in *Haemaphysalis* spp. (Fig 2). Phylogenetic analysis (maximum likelihood method) showed that the 9 sequences in this study belonged to clade B, along with the previously described CLE 16S rRNA sequences of *H*. *longicornis* from Korea (AY342035, AY342036), *H*. *longicornis* from Japan (AB001519), *H*. *longicornis* from China (KC776318, JN866564), and *H*. *lagrangei*, *H*. *obesa*, and *H*. *shimoga* from Thailand (JQ764625, KC170757, KC170759, KC170760) (Fig 2).

## Discussion

The endosymbionts, generally confined to the ovaries and/or the Malpighian tubules of the vectors, have been identified as *Coxiella*, *Wolbachia*, or *Rickettsia* spp. [17]. The relationship between the endosymbionts and the bacteria that are transmitted by ticks and are pathogenic to animals and humans remains unclear [17]. In various arthropods, maternally inherited endosymbionts use adaptive strategies to spread and persevere within host populations, either by skillfully regulating host reproduction or by providing fitness profits to the infected host [5]. Therefore, maternally inherited CLE may be of great ecological and functional importance in tick biology, and may modify their role as vectors of diverse tick-borne pathogens [5].

The finding that ticks can carry both CLE and *C*. *burnetii* emphasizes the need for clear differentiation between them [8]. Various methods to detect *C*. *burnetii* were used in previous studies, and some of those studies offer clear evidence that *Amblyomma tigrinum* and *Dermacentor andersoni* ticks are infected by *C*. *burnetii* rather than by CLE [8]. Several other studies that attempted to determine *C*. *burnetii* prevalence in ticks have not been as accurate, and may have misidentified CLE as *C*. *burnetii* [8]. We previously investigated equine infections in Korea: the seroprevalence of *C*. *burnetii* was 1.3% (11/816) and the presence rate of CLE was 0.7% (6/816) [12]. Therefore, another aim of this study was to assess the differences between the *C*. *burnetii* and CLE found in horse-attached vector ticks by genotyping.

Over the last few decades, climate changes due to global warming have transformed the Korean climate from temperate to subtropical [12]. This may also explain the spread of *Coxiella* in vector ticks in Korea. Most Korean areas are mountainous, and the warm summer season provides suitable conditions for the growth of ticks. From spring to autumn, ticks inhabit the entire country, and their periods of activity are extended [12]. Therefore, the seasonal occurrence frequency of ticks is variable, and the frequency of tick-borne diseases can vary as well. In this study, we showed, using nPCR, that 52.4% (121/213) of the tested ticks infesting horses found on Jeju Island were positive for CLE. The prevalence rate of CLE was considerably higher than that reported within several other studies: 2 of 100 (2.0%) environmental *H*. *longicornis* ticks, collected by flagging vegetation in the Chungbuk province, located in the middle of the Korean Peninsula [18]; 2 of 102 (2.0%) *Rhipicephalus microplus* ticks in Thailand [19]; 3 of 45 (6.7%) ticks in Queensland, Australia [20]; and 1 of 109 (0.9%) *H*. *punctata* ticks in Spain [21] were positive for *Coxiella*. On the other hand, the results of this study were similar to a previous extensive screening study, showing 69.5% (637/916) *Coxiella* positivity in ticks [5]. In that study, 40 of the 58 (69.0%) species of ticks tested positive for *Coxiella*, including the genera of hard ticks (*Rhipicephalus*, *Ixodes*, *Dermacentor*, *Amblyomma*, and *Haemaphysalis*) and soft ticks (*Argas* and *Ornithodoros*) found worldwide, with the samples collected from China representing Asia. None of the 43 CLE genotypes reported in the previous studies is identical to *C*. *burnetii* [5]. A different meta-analysis on the presence of CLE in ticks, presenting molecular evidence based on 16S rRNA sequences, revealed that a minimum of 52 tick species carried CLE, with the detection frequency approaching 100% in numerous cases worldwide, for examples, with samples collected from Japan, Korea, China, and Thailand, which represent Asia [8].

The high percentage of CLE isolated from ticks on Jeju Island may be explained by the fact that Jeju Island is located at the southernmost end of Korea, with a warm oceanic climate, which may promote increased tick distribution [12]. *H*. *longicornis* is the predominant tick species on Jeju Island. A study showed that CLE were detected in 56 of 132 (42.4%) *Haemaphysalis* ticks in Thailand [4], and that the detection did not depend on the tick species, rather it may have been influenced by the origin of the source. For example, female ticks harboring CLE passed the bacteria to their offspring via transovarial transmission, as evidenced by the identification of CLE in eggs [22]. Interestingly, female ticks (59.3%) tended to harbor CLE more frequently than males (21.7%) [4]. The life stage (larva, nymph, and adult) of ticks was an important criterion used to determine the prevalence of CLE [4]. All ticks sampled in this study were adults. Therefore, additional comparative studies, based on the developmental stage, sex of ticks, and collection regions, are needed to clarify this aspect of transmission.

A correlation between the presence of CLE in a tick and the health status of the horse would be quite interesting; however, the health status and presence rates of the horses infested with ticks were not investigated, because ticks were collected irrespective of host condition. This study showed that 42 (82.4%) among 51 horses carried ticks containing CLE. The horses on Jeju Island are typically allowed to graze freely in spacious pastures, but those on the mainland are managed semi-intensively; horses are reared in a confined system with limited grazing time [23]. Thus, horses on Jeju Island are more likely to be exposed to ticks from the natural environment. Interestingly, in a previous study, only 1 horse (1.1%, 1/94) on Jeju Island was detected as CLE positive, while 5 horses (0.7%, 5/722) on the mainland were detected as CLE positive [12]. The low infection rate in horses was not predicted; the infection rates in horses is likely to be high because of high prevalence of ticks at Jeju Island. This may be different for different sampling dates, hosts, breeds, and seasons. Another reason could be that CLE are avirulent and presently pose a much lower infection risk to vertebrates than *C*. *burnetii* [5]. *C*. *burnetii* was not detected in the ticks in this study, and likewise, *C*. *burnetii* was absent in the sera and DNA of horses on Jeju Island [12]. It means that infection with *C*. *burnetii* is rarer than that with CLE in the study region. Additional investigation is warranted to assess the prevalence of *C*. *burnetii* or CLE infection in the ticks and horses reared on Jeju Island at the same time.

Based on phylogenetic analysis, the 9 sequences (HT-14, 17, 24, 65, 122, 131, 157, 220, and 225) analyzed in this study were identical and closely related to the CLE in *Haemaphysalis* ticks, belonging to clade B. These sequences were clustered together with the CLE isolates from Korea, Japan, China, Canada, and Thailand, which may indicate the possibility of an epidemiological connection among these isolates, especially in Asia where *Haemaphysalis* ticks are predominantly distributed, compared to any other region.

To the best of our knowledge, this is the first study to discuss the existence of CLE in zoonotic ticks associated with animals in Korea. The findings of this study suggest that ticks serve as a potential reservoir for transmitting *Coxiella* spp. to other animals and humans via tick bites, although Jeju Island carries a very low risk of *C*. *burnetii* transmission. Further investigations of tick-borne *Coxiella* are necessary for comparison by region (mainland or island), host, tick species, and collection season. Given the high percentage of CLE detected in ticks associated with horses in this study, continuous monitoring as well as implementation of control strategies for wild and domestic animals is necessary. The prevalence of CLE in deer keds suggests the potential involvement of vectors other than ticks, because vectors generally transmit pathogens mechanically or biologically [24]. Moreover, the pathogenicity and risk of zoonotic infections in humans should be considered in all strategies.

## Conclusions

Although relatively little is known about the prevalence of tick-borne *Coxiella* in Korea, it is important to investigate tick infestation risk, especially from the zoonotic as well as public health perspective. CLE were shown to be highly prevalent in ticks that infest horses reared on Jeju Island in Korea; this study, to the best of our knowledge, is the first to describe CLE occurrence in ticks found on animals reared in Korea. Because CLE is highly prevalent in horse-infesting ticks, our findings show that the transmission of *Coxiella* from ticks to other animals and humans following exposure to ticks during horseback riding remains a possibility.
